# Locus of Control and Its Association with Depression, Anxiety, and Stress Among Mexican University Students: A Cross-Sectional Study

**DOI:** 10.3390/ijerph23010130

**Published:** 2026-01-21

**Authors:** Magnolia Guerrero Castillo, Maria Fernanda Martinez Gonzalez, Andrea Alejandra Escalera Jasso, Luis Adrian Alvarez Lozada, Arcelia Lizbeth Torres Pérez, Alejandro Quiroga Garza, Rosa Ivett Guzman Avilan, Diego Escamilla Magaña, Rodrigo Bravo Garcia, Martha Lilia Pérez Sosa, Yelyann Magory Márquez González, Rodrigo Enrique Elizondo Omaña, Guillermo Jacobo Baca

**Affiliations:** 1 Clinical-Surgical Research Group (GICQx), Department of Human Anatomy, School of Medicine, Universidad Autónoma de Nuevo Leon, Monterrey 64460, Nuevo Leon, Mexico; magnolia.guerrerocl@uanl.edu.mx (M.G.C.); luis.alvarezlz@uanl.edu.mx (L.A.A.L.); dr.aquirogag@gmail.com (A.Q.G.); rguzmana@uanl.edu.mx (R.I.G.A.); diego.escamillamgn@uanl.edu.mx (D.E.M.); rodrigo.bravog@uanl.edu.mx (R.B.G.); 2 Centro Universitario de Salud, Universidad Autónoma de Nuevo León, Monterrey 64460, Nuevo León, Mexico; maria.martinezgn@uanl.mx (M.F.M.G.); andrea.escaleraj@uanl.edu.mx (A.A.E.J.); lizbeth.torresprz@uanl.edu.mx (A.L.T.P.); martha.perezs@uanl.mx (M.L.P.S.); yelyann.marquezg@uanl.mx (Y.M.M.G.)

**Keywords:** locus of control, depression, anxiety, stress, mental health, Mexico, university students

## Abstract

Locus of control is a psychological construct that influences how individuals perceive their ability to manage life events. Although its relationship with mental health has been widely studied, limited evidence exists among Latin-American university student populations. To assess the association between locus of control and symptoms of depression, anxiety, and stress among university students in Nuevo León, Mexico. A cross-sectional, analytical study was conducted using data from the Healthy Lifestyle Promotion Program at the Universidad Autónoma de Nuevo León. A total of 815 students completed a digital survey assessing sociodemographic variables, DASS-21, and Burger’s Locus of Control Scale. Participants had a mean age of 19.8 ± 3.4, 57.1% were women. The prevalence of symptoms was 64.4% for depression, 55.8% for stress, and 74.1% for anxiety. Women exhibited higher severity across all dimensions (*p* < 0.001). Locus of control scores decreased significantly as the severity of depression, stress, and anxiety increased, particularly in moderate to extremely severe categories. An external locus of control was consistently associated with higher frequency of severe psychological symptoms. An external locus of control was strongly associated with poorer mental health outcomes. Strengthening internal locus of control may enhance resilience, reduce psychological distress, and improve academic performance.

## 1. Introduction

Locus of control, initially conceptualized by Rotter (1966) [1], refers to the extent to which individuals attribute life events to their own actions (internal locus of control) or to external factors, such as chance, fate, or the influence of others (external locus of control). This psychological construct has been widely studied in the field of mental health and has become a central theoretical framework for understanding how individuals perceive control over life circumstances, cope with stressful situations, and how these perceptions influence emotional well-being, particularly in relation to stress and depression [1,2,3,4].

Individuals with an external locus of control tend to perceive life events and outcomes as beyond their personal control, which makes them more vulnerable to psychological distress, including depression, anxiety, and stress [5,6]. In this context, some studies suggest that this vulnerability may be explained by a personality orientation that increases susceptibility to external influences perceived as uncontrollable, thereby fostering maladaptive responses to stressful situations. Consistently, the available evidence has shown that individuals with an external locus of control are at a higher risk of developing depressive symptoms [3,7,8,9]. By contrast, an internal locus of control has been consistently associated with higher levels of resilience, more effective coping strategies, and better academic performance [10,11].

In this context, locus of control acquires particular relevance in populations exposed to high levels of demand, particularly within the university setting, where students constitute a group especially vulnerable to mental health disorders. Global evidence indicates that approximately one in three university students experience depressive symptoms, while anxiety disorders are among the most prevalent conditions in this age group [12]. Furthermore, university students not only present a higher baseline risk of depressive symptoms but are also exposed to stressors more frequently than other age groups, with reported stress prevalence rates reaching up to 79% [13,14]. In the Latin American context, several studies have shown that factors such as socioeconomic instability, academic overload, and limited access to psychological support services contribute significantly to elevated levels of emotional distress in this population [15,16,17,18]. In this sense, examining the occurrence and correlations of depressive symptoms in response to stressful events within the university environment is particularly relevant for understanding and addressing the determinants of psychological distress in this population.

Despite the extensive evidence generated in Europe, Asia, and North America, research on the role of locus of control in the mental health of university populations in Latin America, particularly in Mexico, remains limited [19]. Sociocultural factors specific to the region, such as family expectations, economic uncertainty, and cultural values, may differentially influence students’ perceptions of control and coping styles compared with other contexts. From a public health perspective, understanding these dynamics is essential for the development of culturally sensitive interventions and institutional policies aimed at promoting mental health, strengthening resilience, optimizing academic performance, and reducing psychological distress among university students.

Furthermore, the use of validated psychometric instruments allows for a systematic assessment of these associations within the Mexican university context. Within this framework, the present study aimed to analyze the association between locus of control and the severity of symptoms of depression, anxiety, and stress among students at one of the largest public universities in Mexico.

## 2. Materials and Methods

### 2.1. Study Design and Setting

An analytical cross-sectional study was conducted using secondary data from the Healthy Lifestyle Promotion Program of the Universidad Autonoma de Nuevo León (UANL), Mexico. Students were invited to participate during institutional activities of the program. This program collects health-related information from students engaged in the Comprehensive Training Activities through structured educational sessions designed to promote healthy behavior. Data were obtained from a single cross-sectional assessment conducted in June 2025 through this program. The study was reported in accordance with the Strengthening the Reporting of Observational Studies in Epidemiology (STROBE) guidelines [20].

### 2.2. Study Population

The study population comprised high school and bachelor’s students enrolled in any of the UANL campuses at the time of the study. Participation was entirely voluntary, and responses were collected anonymously via an online questionnaire (Google Forms). The survey included an explicit informed consent form, detailing the study objectives, its significance, and participants’ rights. A non-probabilistic convenience sampling method was employed; therefore, the possibility of selection bias cannot be entirely ruled out. Nevertheless, the inclusion of students from multiple academic fields and educational levels contributed to the heterogeneity of the sample. Eligibility criteria included: (1) complete responses to the digital survey, and (2) valid, non-duplicate records. Students with incomplete or inconsistent data were excluded.

### 2.3. Variables and Instruments

#### 2.3.1. Burger’s Locus of Control Scale

The primary exposure variable was locus of control, assessed using Burger’s Locus of Control Scale [21]. This self-report instrument consists of 10 items and yields a total score ranging from 10 to 70. Higher scores indicate an internal orientation, reflecting that the individual perceives having active control over life events; conversely, lower scores indicate an external orientation, suggesting that the individual perceives life outcomes as being determined by factors beyond their control. Each item is rated on a 7-point Likert scale, ranging from 1 (strongly disagree) to 7 (strongly agree), and responses are summed to generate an overall measure of perceived control over life events.

#### 2.3.2. Depression, Anxiety, and Stress Scales (DASS-21)

Mental health outcomes were assessed using the 21-item version of the Depression, Anxiety, and Stress Scales (DASS-21), an abbreviated form of the original 42-item scale, designed to assess self-report levels of depression, anxiety, and stress in adolescents and young adults [22]. Each subscale measures a specific psychological construct: depression reflects symptoms associated with low mood, lack of motivation, and feelings of hopelessness; anxiety encompasses symptoms of physiological arousal, fear, and tension; and stress focuses on symptoms of tension, irritability, and difficulty relaxing.

The scale comprises 21 items, with seven items per subscale. Each item is rated on a 4-point Likert scale ranging from 0 to 3, where 0 indicates that the statement “does not apply at all,” 1 indicates that it “applied to me to some degree, or some of the time,” 2 indicates that it “applied to me to a considerable degree, or a good part of time,” and 3 indicates that it “applied to me very much, or most of the time.” The raw score for each subscale ranges from 0 to 21, and the scores are classified into severity levels as follows: for depression, no symptoms (0–6), mild (7–8), moderate (9–13), severe (14–16), and extremely severe (≥17); for anxiety, no symptoms (0–5), mild (6–7), moderate (8–12), severe (13–15), and extremely severe (≥16); and for stress, no symptoms (0–11), mild (12–13), moderate (14–16), severe (17–18), and extremely severe (≥19)

This scale allows for systematic assessment of the severity of emotional symptoms and their relationship with other psychological variables, such as locus of control, in university students and young adult populations.

Sociodemographic covariates included age, sex, educational level, occupation, living arrangement, marital status, and academic field.

### 2.4. Statistical Analysis

Descriptive statistics were used to characterize the sample, expressed as means and standard deviations for continuous variables, and frequencies with percentages for categorical variables. Differences between men and women were assessed using chi-square tests for categorical data and *t*-tests or Mann–Whitney U tests for continuous variables, depending on normality assumptions. Normality was assessed using the Kolmogorov–Smirnov test.

To examine the association between locus of control and symptom severity, Kruskal–Wallis tests and pairwise comparisons with 95% confidence intervals were performed. A *p*-value < 0.05 was considered statistically significant. Analyses were performed using IBM SPSS Statistics version 26.0.

### 2.5. Ethical Considerations

The study was conducted in accordance with the principles of the Declaration of Helsinki and the provisions of the General Health Law of Mexico on Health Research. As the analysis was performed using previously anonymized data, it was classified as minimal risk. The study protocol was reviewed and approved by the Ethics and Research Committees of the Hospital Universitario Dr. José Eleuterio González (approval number: AH25-00003). All data were handled with strict confidentiality.

## 3. Results

A total of 815 students were included in the analysis, with a mean age of 19.8 ± 3.4 years, 57.1% of the sample were female. Most participants were enrolled in bachelor’s degree programs (74.5%), lived with their families (90.1%), and reported not having a romantic partner (62.3%). The most represented academic field was Social and Administrative Sciences (46.6%), followed by high school students (22.7%) (Table 1).

Across the DASS-21 dimensions, the prevalence of psychological symptoms was high. Depressive symptoms were reported by 64.4% of participants, stress symptoms by 55.8%, and anxiety symptoms by 74.1%, with mean scores of 7.28 ± 5.18, 7.55 ± 4.87, and 7.05 ± 4.85, respectively. Women exhibited significantly higher mean scores and a greater proportion of cases classified as moderate, severe, and extremely severe across all dimensions (*p* < 0.001) (Table 2). In contrast, no significant sex differences were observed in locus of control scores (*p* = 0.979).

**Figure 1:**
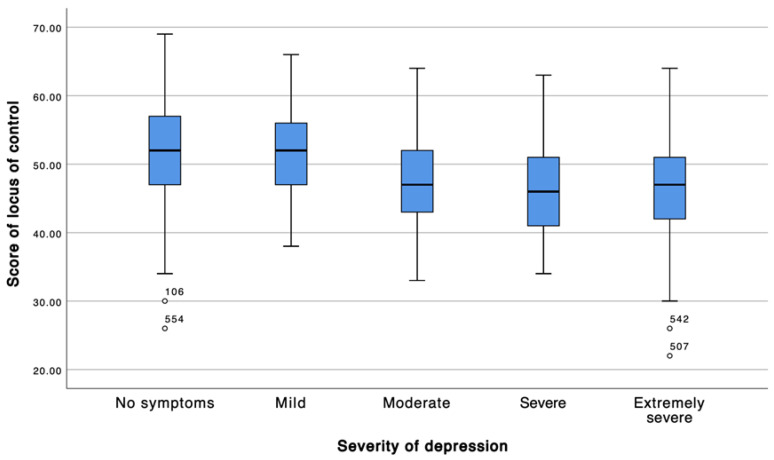
According to severity of depression. The box represents the interquartile range (IQR) and the horizontal line indicates the median. Whiskers extend to 1.5 × IQR. Note: White circles indicate outliers (values outside 1.5 × IQR); labels correspond to the participant ID.

**Figure 2:**
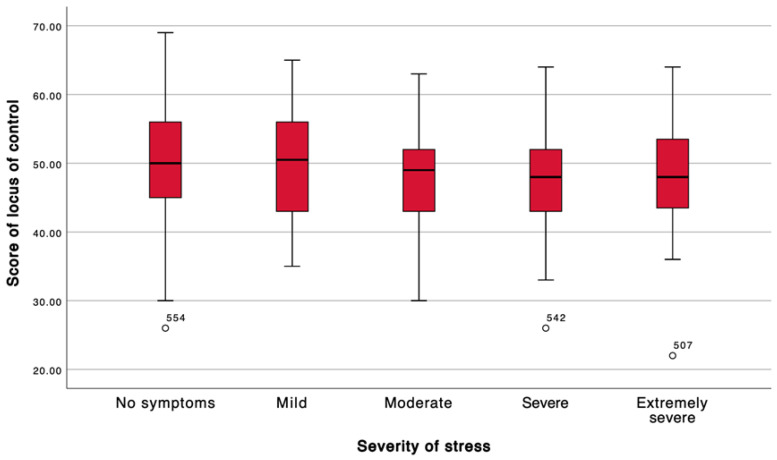
According to severity of stress. The box represents the interquartile range (IQR) and the horizontal line indicates the median. Whiskers extend to 1.5 × IQR. Note: White circles indicate outliers (values out-side 1.5 × IQR); labels correspond to the participant ID.

**Figure 3:**
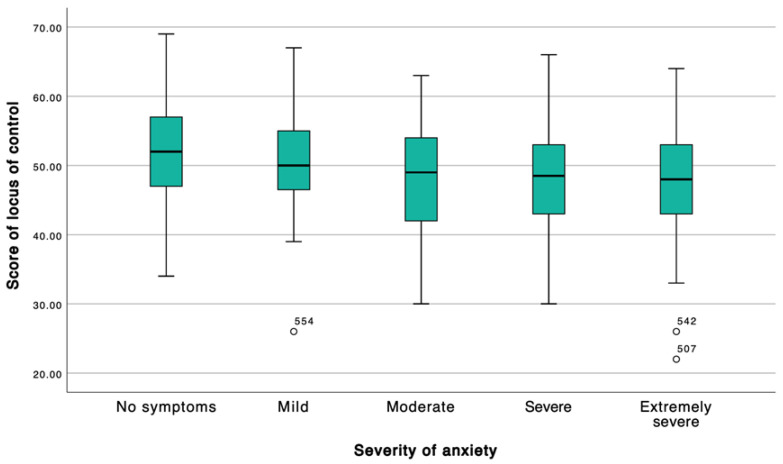
According to severity of anxiety. The box represents the interquartile range (IQR) and the horizontal line indicates the median. Whiskers extend to 1.5 × IQR. Note: White circles indicate outliers (values out-side 1.5 × IQR); labels correspond to the participant ID.

Locus of control was inversely associated with symptom severity. Students without symptoms exhibited the highest locus of control scores, whereas those classified as having moderate, severe, or extremely severe symptoms demonstrated significantly lower scores. This graded pattern was consistently observed across the depression, stress, and anxiety dimensions, with the most pronounced differences occurring between asymptomatic participants and those in the severe symptom categories (Table 3; Figure 1, Figure 2 and Figure 3).

## 4. Discussion

This study investigated the association between locus of control and mental health symptoms in a large sample of Mexican university students. The findings indicate that an external locus of control is associated with greater severity of depressive, anxiety, and stress-related symptoms, whereas an internal locus of control appears to confer a protective effect. Collectively, these results underscore the relevance of locus of control as a salient psychological determinant of emotional well-being among young adults.

The high prevalence of psychological symptoms observed in this sample is consistent with prior reports from Latin America. In Mexico, university students frequently report elevated levels of anxiety and depression, which are commonly attributed to academic demands, socioeconomic uncertainty, and limited access to mental health services [15,16]. Consistent with international trends, women in the present study exhibited significantly greater symptom severity, aligning with evidence indicating that female students are more susceptible to internalizing disorders, potentially because of the interplay between biological, psychosocial, and sociocultural factors [23,24].

The observed inverse association between locus of control and symptom severity is consistent with findings reported in other regions [25]. Individuals with an external locus of control are more likely to attribute adverse events to uncontrollable external forces, a tendency that may exacerbate feelings of helplessness and heighten vulnerability to emotional distress [2,5]. In contrast, students who perceive greater control over life events are more inclined to employ adaptive coping strategies and sustain a stronger sense of self-efficacy, thereby promoting psychological resilience and supporting academic functioning [10,26].

With respect to depressive symptomatology, Khumalo et al. (2019) reported, in a study conducted among university students using multiple regression analysis, that individuals who attributed life events to chance or luck tended to exhibit higher levels of depression, whereas an internal locus of control appeared to function as a protective factor [5]. These findings are consistent with those observed in the present study, in which participants with higher levels of depression demonstrated lower mean scores on the Burger Locus of Control Scale, reflecting a more externally oriented locus of control, while those without depressive symptoms exhibited higher scores on this instrument. This association may be explained by the tendency of individuals with an external locus of control to perceive life events as independent of their own actions, which fosters feelings of helplessness, low self-efficacy, and hopelessness, reduces the use of active coping strategies, and consequently increases vulnerability to depressive symptomatology [5,27,28,29].

In the present study, participants with higher levels of depression not only exhibited a more externally oriented locus of control, but this pattern was also observed across stress and anxiety levels, particularly in the more severe categories of anxiety. These findings are consistent with those reported by Wenzhen et al. (2025), who identified an association between externality attributed to chance and a higher prevalence of anxiety and depression [25]. This relationship may be explained by the perception of limited control over personal outcomes, which fosters passive and pessimistic attitudes and generates feelings of helplessness, thereby increasing vulnerability to anxiety and depressive symptoms [30,31].

Additionally, the role of health risk behaviors in the relationship between health-related locus of control and disorders has been explored, underscoring their relevance for the design of interventions targeting university populations [32,33]. This is particularly pertinent given the positive association described between an external locus of control and increased symptoms of anxiety and depression when such risk behaviors are not adequately addressed [25].

No significant sex differences in locus of control were observed, a finding consistent with previous studies [34]. This suggests that the association between locus of control and mental health outcomes may operate largely independently of sex. Instead, sociocultural factors—such as living arrangements, relationship status, and economic dependence—may exert a more substantial influence on the formation of control perceptions [35,36]. Future research should examine these contextual variables in greater depth to more clearly characterize the mechanisms underlying psychological vulnerability within student populations.

From an applied perspective, the findings of the present study suggest that locus of control may be considered a relevant psychological variable for the early identification of students at increased emotional vulnerability [2,8]. Although it does not constitute a diagnostic tool, its consistent association with the severity of depression, anxiety, and stress symptoms supports its potential utility as a complementary screening instrument in university settings, particularly within mental health promotion and prevention programs [9,17,25].

It is important to note that locus of control is not a homogeneous construct and that different approaches to its measurement exist. Unidimensional scales, such as those developed by Rotter or Burger, assess the internal–external continuum and are particularly suitable for large-scale population studies due to their brevity and ease of administration. In contrast, multidimensional instruments, such as Levenson’s scale and other scales, allow for the differentiation of multiple sources of external control, providing a more nuanced assessment of the construct at the expense of greater methodological complexity [37,38,39,40]. In this context, the use of brief scales may be especially appropriate in university environments; however, additional longitudinal studies are required to confirm their predictive value and their utility in early prevention and intervention strategies [5,32].

Our findings highlight the importance of interventions aimed at strengthening an internal locus of control as a strategy to reduce psychological vulnerability among students. In university settings, this may be achieved through initiatives such as decision-making skills training, resilience-building programs, and the provision of accessible psychological support services. Evidence suggests that enhancing internal control not only protects against anxiety and depression but also contributes to higher self-esteem, motivation, and academic performance [11,12].

From a mental health promotion perspective, the findings of this study provide relevant evidence to inform the design and targeting of university-based programs aimed at enhancing psychological well-being. Such interventions may focus on strengthening an internal locus of control as a strategy to reduce psychological vulnerability among students, as well as on prioritizing preventive interventions for individuals with an external locus of control, who are at greater risk of experiencing more severe psychological symptoms [13,27].

Within university settings, these strategies could be implemented through initiatives such as decision-making skills training, programs aimed at enhancing resilience and self-efficacy, and the provision of accessible psychological support services. Evidence suggests that fostering a stronger internal sense of control not only protects against anxiety and depression but also contributes to higher levels of self-esteem, motivation, and academic performance [11,12].

## 5. Conclusions

This study identified that an external locus of control is associated with greater severity of depressive, anxiety, and stress symptoms among Mexican university students, whereas an internal locus of control is related to higher emotional well-being. These findings provide relevant empirical evidence regarding the importance of locus of control as a psychological factor closely linked to mental health in a particularly vulnerable population and highlight its potential utility as a modifiable resource within the framework of psychological well-being promotion.

In this context, the results underscore the value of implementing university-based strategies aimed at strengthening perceived control, such as promoting autonomous decision-making, fostering self-efficacy, and improving access to psychological support services. Nevertheless, these approaches should be regarded as complementary rather than substitutive to clinical assessment and specialized care, and their effectiveness should be examined through longitudinal and interventional studies.

### 5.1. Limitations

This study has several limitations that should be acknowledged. First, the cross-sectional design precludes the establishment of causal relationships between locus of control and psychological symptoms. Second, reliance on self-reported measures may introduce social desirability and reporting biases. Third, the heterogeneity of the sample, including students from both high school and university levels, as well as from diverse academic disciplines—may limit the generalizability of the findings. Nevertheless, the large sample size and the use of robust statistical analyses enhance the reliability and validity of the results.

### 5.2. Future Research

Future research should employ longitudinal designs to clarify the causal relationships between locus of control and mental health outcomes. Prospective cohort studies would be valuable for examining the longitudinal associations between health risk behaviors and the different dimensions of locus of control, as well as their role in the development and progression of other mental health disorders. Additionally, intervention studies aimed at strengthening an internal locus of control and enhancing adaptive coping skills are warranted to determine whether such approaches effectively reduce psychological distress in university settings. Finally, the incorporation of socioeconomic variables and coping strategies may provide a more comprehensive understanding of psychological vulnerability and resilience among Latin American student populations.

## Figures and Tables

**Table 1:** Sociodemographic Characteristics.

Characteristic	*n* (%)
Sex	
Women	466 (57.1)
Men	345 (42.3)
Other	4 (0.6)
Current degree	
High School	208 (25.5)
College	607 (74.5)
Occupation	
Exclusively studying	516 (63.3)
Studying and occasionally working	131 (16.1)
Studying and regularly working	168 (20.6)
Living status	
Family	734 (90.1)
Roommates	27 (3.3)
Couple	20 (2.5)
Alone	34 (4.2)
Romantic relationship	
With a partner	307 (37.7)
Without a partner	508 (62.3)
Field of Knowledge	
Arts, Education, and Humanities	67 (8.2)
Agricultural Sciences	4 (0.5)
Health Sciences	44 (5.4)
Natural and Exact Sciences	64 (7.9)
Social and Administrative Sciences	380 (46.6)
Engineering and Technology	71 (8.7)
High School	185 (22.7)
*n*: sample; %: percentage	

**Table 2:** Differences between men and women in DASS-21 dimensions and locus of control.

	General *n* = 815	Men *N* = 345 (42.3)	Women *N* = 465 (57.1)	*p*-Value
**Depression (mean ± SD)**	7.28 ± 5.18	6.34 ± 4.76	7.90 ± 5.37	<0.001 *
No symptoms	290 (35.6)	143 (41.4) ^a^	147 (31.6) ^b^	0.001 *
Mild	103 (12.6)	48 (13.9) ^a^	55 (11.8) ^a^	
Moderate	217 (26.6)	91 (26.4) ^a^	126 (27.1) ^a^	
Severe	82 (10.1)	29 (8.4) ^a^	50 (10.8) ^a^	
Extremely Severe	123 (15.1)	34 (9.9) ^a^	87 (18.7) ^b^	
**Stress (mean ± SD)**	7.55 ± 4.87	7.48 ± 4.39	9.31 ± 5.08	<0.001 *
No symptoms	360 (44.2)	177 (51.3) ^a^	183 (39.4) ^b^	<0.001 *
Mild	132 (16.2)	59 (17.1) ^a^	72 (15.5) ^a^	
Moderate	150 (18.4)	64 (18.6) ^a^	84 (18.1) ^a^	
Severe	118 (14.5)	37 (10.7) ^a^	79 (17) ^b^	
Extremely Severe	55 (6.7)	8 (2.3) ^a^	47 (10.1) ^b^	
**Anxiety (mean ± SD)**	7.05 ± 4.85	5.92 ± 4.25	7.84 ± 5.10	<0.001 *
No symptoms	211 (25.9)	112 (32.5) ^a^	99 (21.3) ^b^	<0.001 *
Mild	67 (8.2)	35 (10.1) ^a^	32 (6.9) ^a^	
Moderate	201 (24.7)	90 (26.1) ^a^	111 (23.9) ^a^	
Severe	98 (12)	35 (10.1) ^a^	63 (13.5) ^a^	
Extremely Severe	238 (29.2)	73 (21.2) ^a^	160 (34.4) ^b^	
**Locus of control (mean ± SD)**	49.26 ± 7.33	49.39 ± 7.92	49.25 ± 6.84	0.979

a, b—Sharing superscript denotes similarity by Z-test with Bonferroni adjustment for multiple comparisons. The DASS-21 scale ranges from 0 to 21 for each dimension, while the locus of control scale ranges from 10 to 70. Different superscript letters indicate statistically significant differences (*: *p* < 0.05) between men and women. SD: standard deviation; values in parentheses expressed as percentages.

**Table 3:** Locus of control scores by severity categories.

**Depression**	**No** **Symptoms**	**Mild**	**Moderate**	**Severe**	**Extremely Severe**
No symptoms		0.24 [−1.95, 2.43]	3.89 * [2.17, 5.6]	4.97 * [2.58, 7.36]	5.32 * [3.27, 7.37]
Mild			3.65 * [1.36, 5.93]	4.73 * [1.91, 7.55]	5.08 * [2.53, 7.63]
Moderate				1.08 [−1.39, 3.56]	1.43 [−0.72, 3.59]
Severe					0.35 [−2.37, 3.07]
**Stress**	**No** **symptoms**	**Mild**	**Moderate**	**Severe**	**Extremely severe**
No symptoms		0.39 [−1.63, 2.42]	1.94 * [0.01, 3.87]	2.43 * [0.32, 4.54]	2.08 [−0.80, 4.96]
Mild			1.55 [−0.83, 3.92]	2.03 * [−0.48, 4.55]	1.69 [−1.50, 4.88]
Moderate				0.49 [−1.96, 2.93]	0.14 [−2.99, 3.28]
Severe					0.35 [−3.59, 3.59]
**Anxiety**	**No** **symptoms**	**Mild**	**Moderate**	**Severe**	**Extremely severe**
No symptoms		1.25 [−1.49, 4.00]	3.11 [1.18, 5.04]	3.71 * [1.32, 6.11]	4.00 * [2.15, 5.85]
Mild			1.86 [−0.91, 4.62]	2.46 * [−0.64, 5.56]	2.75 * [0.04, 5.46]
Moderate				0.61 [−1.80, 3.02]	0.89 [−0.98, 2.77]
Severe					0.29 [−2.06, 2.06]

Note: Asterisks (*) indicate statistically significant differences after Bonferroni adjustment (*p* < 0.05). Normality was rejected (KS test, *p* = 0.002); Mann–Whitney U tests were applied for comparisons.

## Data Availability

The data presented in this study are available on request from the corresponding authors.
